# A cautionary TALE: how plant breeding may have favoured expanded TALE repertoires in *Xanthomonas*

**DOI:** 10.1111/mpp.12670

**Published:** 2018-05-03

**Authors:** Niklas Schandry, Jonathan M. Jacobs, Boris Szurek, Alvaro L. Perez‐Quintero

**Affiliations:** ^1^ Gregor Mendel Institute (GMI), Austrian Academy of Sciences, Vienna BioCenter (VBC) Vienna 1030 Austria; ^2^ Department of Bioagricultural Sciences and Pest Management Colorado State University Fort Collins CO 80523 USA; ^3^ Applied Microbiology ‐ Phytopathology Earth and Life Institute, Université Catholique de Louvain Louvain‐la‐Neuve, 1348 Belgium; ^4^ IRD, Cirad, University of Montpellier, IPME Montpellier 34000 France; ^5^ Institut de Biologie de l'Ecole Normale Supérieure (IBENS), Ecole Normale Supérieure, CNRS, INSERM, PSL Research University Paris 75005 France

The gammaproteobacterial genus *Xanthomonas* contains several plant‐pathogenic species. Many economically important crops serve as hosts for different *Xanthomonas* spp., making this genus a considerable threat to global agriculture and food security. Rice‐pathogenic *X. oryzae* pathovars *oryzae* (*Xoo*) and *oryzicola* (*Xoc*) cause bacterial blight (BB) and bacterial leaf streak (BLS), respectively, with major losses for farmers in the tropics. Other important pathogens in this genus include *X. euvesicatoria*, *X. gardneri* and *X. perforans* species that infect solanaceous plants, various *X. translucens* pathovars that infect wheat, barley and other grasses, *X. citri* ssp. *citri* (*Xci*), the causal agent of citrus canker, *X. campestris* pv. *campestris*, the causal agent of black rot disease of Brassicaceae, and *X. citri* ssp. *malvacearum* (*Xcm*) and *X. phaseoli* pv. *manihotis* (*Xpm*), which cause BB of cotton and cassava, respectively (Jacques *et al*., 2016).

All of the above‐mentioned *Xanthomonas* pathogens rely on the use of transcription activator‐like effectors (TALEs) to infect host plants. TALEs are type III‐secreted proteins that act as modular, sequence‐specific transcription factors inside plant cells. The N‐ and C‐terminal regions of these proteins contain domains that allow for translocation into plant cells (N‐terminal type III secretion signal), import into the nucleus (C‐terminal nuclear localization signal) and their interaction with the eukaryotic transcriptional machinery (C‐terminal acidic activation domain).

TALEs also harbour a central repeat domain (CRD), which confers their sequence‐specific DNA binding ability. The CRD is made up of nearly identical 33–35‐amino‐acid repeat sequences that differ primarily at positions 12 and 13, called the repeat variable diresidue (RVD). Each repeat binds one single DNA base and base specificity is determined by the RVD. As the CRD domain confers DNA targeting specificity, alterations in repeat number or the order of RVDs result in different targeting specificities (Boch and Bonas, 2010).

TALEs bind to the promoter regions of so‐called susceptibility ‘*S*’ genes. Subsequent TALE‐triggered *S* gene induction promotes pathogenesis and enhances bacterial multiplication for disease development. Examples of *S* genes include *SWEET*s, which encode individual members of a family of sugar transporters. All four species *Xoo*, *Xci, Xpm* and *Xcm* induce *SWEET*s in a TALE‐dependent manner in rice, citrus, cassava and cotton, respectively. *SWEET* induction is hypothesized to induce extracellular sugar export to provide a nutrient source for pathogen multiplication (Hutin *et al*., 2015).

At least three other species from different bacterial genera harbour TALE‐like proteins: *Ralstonia solanacearum*, *Paraburkholderia rhizoxinica* and one or more undetermined species of marine bacteria. Only the TALE‐like proteins of the solanaceous pathogen *R. solanacearum* (called RipTALs) carry N‐ and C‐terminal domains that allow them to act as bona fide transcription factors; however, a clear role in virulence has not yet been assigned to these proteins (Schandry *et al*., 2016).

**Figure 1:**
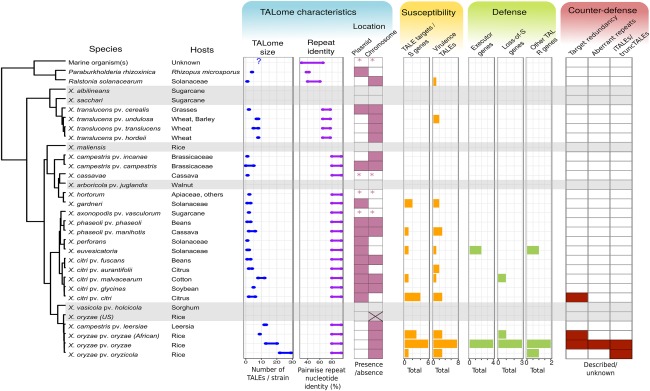
Summary of TALome characteristics and transcription activator‐like effector (TALE)‐related offence and defence strategies (from left to right). The tree shows phylogenetic relations of various *Xanthomonas*, together with other TALE‐like carrying bacteria, based on average nucleotide identity. Taxa highlighted in grey indicate cases in which no TALE was found in the currently available genomic data; in the other taxa, at least one strain is known to carry at least one TALE. The preferred hosts for each bacterium are shown. The characteristics of TALEs from these groups (TALome size, repeat identity or location) were obtained from the literature or from genomic sequences in the National Center for Biotechnology Information (NCBI) gene bank. The range of pairwise repeat nucleotide identity was calculated from representative strains in each group. An asterisk indicates cases in which the genomic location for some TALEs could not be determined as they are found in draft genome contigs or metagenomic sequences. ‘X’ indicates the presence of TALE remnant sequences. The ‘Susceptibility’ and ‘Defence’ bar plots indicate the number of reported occurrences of these strategies for each group. Virulence TALEs include all unique TALEs for which a role in virulence has been proposed, including those TALEs for which no *S* gene targets have been found, but an associated phenotype has been reported. The ‘Counter‐defence’ bar indicates whether any of the mechanisms have been reported in each group. Some TALE‐containing groups were omitted from the figure for brevity or insufficient genomic data, including *X. alfalfae*, *X. theicola*, *X. hyacinthi* and *X. citri* pv. *mangiferaeindicae* (Jacques *et al*., 2016).

The TALE repertoire varies greatly between different *Xanthomonas* pathovars or subspecies, and even between different strains (Fig. 1). Some taxa, such as *X. albilineans*, *X. sacchari* and *X. vasicola*, do not have TALEs. Most groups contain between one and five TALEs, including *X. euvesicatoria* and *Xci*. ‘Medium’‐sized TALomes (5–8) can be found in some *X. translucens*. In contrast, a remarkable expansion in TALome size can be found in *Xanthomonas citri* pv. *mangiferaeindicae* (up to 10), *Xcm* (up to 12) and *X. oryzae*, ranging from nine TALEs per genome in African strains of *Xoo* to 29 in some Asian strains of *Xoc*. Curiously, North American strains of *X. oryzae* do not seem to contain any TALEs; incomplete (probably untranslated) TALE‐like sequences can, however, be found in their genomes, suggesting that these effectors were lost in this group (Fig. 1).

Genome sequencing studies have highlighted that not only is the number of TALEs variable, but also individual TALEs differ in the composition of their CRD and therefore in their DNA binding specificities. One may assume that the large TALome size is necessary to achieve virulence and cause disease. However, this is usually not the case. In many studies aiming to identify the virulence targets of TALEs, researchers have demonstrated that a large fraction of TALomes cannot be linked to a specific virulence phenotype. Instead, only a few TALEs per TALome are virulence factors, and the role of the remaining TALEs remains unclear. This raises the question: why are these large genes encoding potentially costly large proteins retained in the genomes of some *Xanthomonas* lineages? In this article, we hypothesize that diversity in TALome size and composition could result from plant breeding programmes and the rapid deployment of resistant cultivars, particularly in rice‐infecting xanthomonads.

Many *Xanthomonas* hosts, including rice, wheat, citrus, cotton and solanaceous crops, are all subject to intensive breeding programmes. These programmes aim to create disease‐resistant crop varieties, but are often conducted with limited geographical scope. For example, rice is extensively bred in Asian countries, in particular China, where rice breeding has a long history. In contrast, rice breeding in African countries has a comparatively short history. Rice, an annual plant, has short deployment cycles. The breeding and deployment of perennial *Xanthomonas* host species, such as citrus, are slower, simply because these plants have a longer life cycle. Different intensities of breeding may be reflected in the different sized TALomes of rice‐infecting *X. oryzae* strains from different groups, in relation to the diversity of resistance genes identified in rice against TALEs from these groups (Fig. 1).

Resistance genes specific to TALE‐harbouring strains have been identified to date in solanaceous plants, cotton and rice. They can be divided into those that do or do not rely on the activity of TALEs as transcriptional activators. In the first case, resistance against TALEs can be achieved by exploiting their transcriptional activity, ‘tricking’ them into inducing genes that will prevent bacterial colonization. These so‐called executor *R* genes include two genes from pepper (*Bs3* and *Bs4‐C*) and at least four different genes from rice (*Xa7*, *Xa10*, *Xa23* and *Xa27*) (Zhang *et al*., 2015).

Alternatively, the plant can avoid TALE activity altogether by selecting for loss of susceptibility through promoter variation. During loss of susceptibility, nucleotide variation through indels or individual substitutions in promoters does not permit TALE binding, thereby abolishing gene activation and thus preventing disease development. At least three cases of loss of susceptibility have been described in rice (*xa13*, *xa25* and *xa41*; Hutin *et al*., 2015) and one more recently in cotton.

On the other hand, to exert resistance against TALEs independent of their activity, plants can deploy ‘traditional’ *R* genes that rely instead on direct or indirect recognition of the partial structure of TALEs and the subsequent triggering of defence. To date, *R* genes that use this mechanism include the NLR genes *Bs4* from tomato and *Xa1* from rice, and the uncharacterized *Xo1* gene from rice (Zhang *et al*., 2015).

Finally, a very specific type of resistance against TALEs found in rice is represented by the recessive resistance allele *xa5*, which encodes for a variant of TFIIAɣ (a subunit of a transcription factor). Plants homozygous for *xa5* resist TALE‐bearing strains by weakening their interaction with the transcriptional activation machinery (Yuan *et al*., 2016).

From a breeding perspective, the introgression of *R* genes dependent on TALE transcriptional activity (executor gene or loss‐of‐susceptibility alleles) into elite lines is very attractive. This type of resistance avoids the negative impacts that could be associated with ‘traditional’ *R* genes. Often, increased expression of defence pathways by *R* genes has undesired effects, as defence sequesters resources, such as carbohydrates and water, which are also essential for plant development and growth.

However, bacteria can overcome TALE‐specific resistance. For example, *xa5*‐mediated resistance is overcome by TALE‐mediated induction of alternative subunits of TFIIA which are functionally redundant. Further, to avoid the effect of loss‐of‐susceptibility alleles, strains may carry functionally redundant TALEs that bind to multiple sites in the promoters of the same (or related) *S* genes. This seems to be the case for various *Xoo* strains that carry multiple SWEET‐inducing *tale* genes and thus overcome loss‐of‐susceptibility alleles, such as *xa41* and *xa13* (Hutin *et al*., 2015). A more sophisticated mechanism to overcome loss‐of‐susceptibility alleles involves the use of aberrant repeats that introduce flexibility to the TALE structure and allow them to bind to promoter variants of *S* genes that would normally be resistant to induction (e.g. SWEET‐inducing PthXo1 and AvrXa7) (Hutin *et al*., 2015).

In the case of executor genes, it is expected that the TALEs that activate them are rapidly lost in populations, and this seems to be the case for some non‐essential and poorly conserved TALEs (e.g. AvrXa10 and AvrXa27) (Vera‐Cruz *et al*., 2000). Some executor (e.g. *Xa7*) binding TALEs, such as AvrXa7, may persist in the population if they also activate *S* genes essential for pathogenicity (Vera‐Cruz *et al*., 2000).

*Xanthomonas oryzae* strains overcome recognition by traditional *R* genes, *Xo1* and *Xa1*, by deploying structural variants of TALEs that seem to interfere with *R* gene function, possibly by competitively binding the R protein without eliciting an immune response (Zuluaga *et al*., 2017). It is conceivable that the loss of TALEs in some US strains is a response to the widespread presence of these *R* genes in cultivated rice in North America. Consequently, these strains avoid recognition and resistance at the expense of a significant loss of virulence.

We believe that these described counter‐defence mechanisms are a testament to the effect of the deployment of *R* genes on TALome evolution. Indeed, to date, these mechanisms have been found almost exclusively in *X. oryzae* strains with expanded TALomes (Fig. 1). We propose that the expansion of TALomes is thus a feature that has been selected for in response to plant resistance, as it allows the bacteria to develop counter‐defence strategies and to quickly adapt to new cultivars.

On deployment in the field, resistant varieties exert a selection pressure on the resident pathogen population to overcome or evade resistance (Vera‐Cruz *et al*., 2000). As one possible evolutionary route to secure their ecological niche in the context of rapidly bred plants, the pathogens potentially accelerate the rate of evolution of their own virulence arsenal through increased evolvability of the genes. TALEs and, in particular, the CRD, exhibit some hallmarks of evolvability as defined by Kirschner and Gerhart (1998): modularity and weak linkage. As mentioned above, TALEs are modular and divided into N‐terminal, C‐terminal and CRD modules. The CRD itself is composed of modular repeats with each being independent in function of the other. However multiple repeats in concert give rise to a functional module, providing a second layer of modularity.

Weak linkage, a term used to describe functional independence from other cellular processes, is also found in TALEs. TALEs depend on the pathogen's type III secretion system for secretion, but there are no described dependences on other effectors. Within the host, TALEs interact with the transcriptional machinery, which is mostly conserved between plant species. It is notable that the TALE CRD exhibits both increased modularity and exceptionally weak linkage, as the protein–protein interactions are mediated by the N‐ and C‐terminal regions of TALEs.

From a genetic and genomic perspective, *TALEs* are unstable genes. As a result of the repetitive character of the CRD, single TALEs are genetically unstable and prone to recombinatorial mutation. When comparing *R. solanacearum* RipTALs and *Xanthomonas* TALEs, the repeats of the latter group are generally less diverse, as evidenced by higher pairwise nucleotide identity (Fig. 1). We have previously hypothesized that the decrease in repeat diversity in *Xanthomonas* reflects a selection for evolvability, as more similar repeats are more likely to recombine. This could explain why *Xanthomonas* species in general seem to be more reliant than *R. solanacearum* on TALEs for infection (Schandry *et al*., 2016).

In *Xanthomonas*, frequent recombination of repeats may increase the rate of stochastic phenotype switching through altered DNA binding specificities and, consequently, increase the adaptive potential of TALEs in the context of changing host genotypes (Kussell and Leibler, 2005). It is also notable that bacterial recombination usually increases under stress (Bjedov *et al*., 2003), suggesting that encountering a resistant cultivar could increase the mutation rate in the given pathogen population, enabling rapid radiation and consequent adaptation. Assuming that a large TALome leads to a higher frequency of phenotype switching in the context of host specificity, the deployment of a new cultivar that exploits TALE‐mediated resistance will exert a strong selection for a specific, switched TALome state from the population. This particular bacterial strain would then have a great selective advantage in this new environment, and rapidly emerge and form a new pathogenic population. Genomic and epidemiological data from *X. oryzae* suggest that these rapid population shifts occur in *X. oryzae* accompanied by changes in TALE repertoires (Vera‐Cruz *et al*., 2000).

Larger TALomes with increased evolvability may then have inadvertently been selected for by attempts to control *Xanthomonas* infections through the regular deployment of new cultivars that are resistant to the current dominant bacterial isolate. This, we believe, is the case for *X. oryzae* strains and, possibly, even for some groups with somewhat expanded TALomes, such as *Xcm* and the *X. translucens* pathovars *undulosa* and *translucens*. They all represent pathogens of hosts with a long history of breeding for resistance.

In the case of African *Xoo* versus Asian *Xoo*, differences in TALome size could indeed partially be explained by the different practices associated with rice domestication and history of cultivation in these two continents, with the African strains keeping an ‘ancestral’ medium‐sized TALome suited to the colonization of the less bred African rice varieties. It is well known that the domestication of *Oryza sativa* in Asia occurred much earlier than that of *Oryza glaberrima* in Africa, where this crop has consequently been cultivated for a shorter time and on less extensive surfaces, which implies less disease pressure and therefore probably less breeding for resistance. To our knowledge, there are no reliable archaeological records of what could be interpreted as testimonies of ancient bacterial epidemics on rice. Yet, reports suggest that devastating epidemics caused by the rice blast fungus *Magnaporthe oryzae* may have occurred in China about 2000 years ago, urging farmers to introduce new varieties that were imported from distant regions. This is in line with the hypothesis that land cultivated with rice was already quite important over 2000 years ago in China. In contrast, rice cultivation in Africa is believed to have been sporadic until the Green Revolution, which was associated with the deployment of intensive cropping of *O. sativa* varieties across the continent. It will be interesting to follow the evolution of *Xoo* TALomes over the next 50 years in sub‐Saharan Africa, a region in which rice production is likely to undergo tremendous changes as a result of the introduction of resistant elite varieties and intensive crop management practices with strong environmental and genetic uniformity.

Curiously, when comparing Asian *Xoo* with *Xoc*, the latter group has generally larger TALomes despite historically facing less breeding and less *R* genes against its TALEs. It is currently unknown when these two lineages diverged or when the expansion occurred, but it is reasonable to believe that their ancestor already possessed a large TALome and had already encountered resistance in the host.

Why then did *Xoc* TALomes increase? It is possible that *Xoc* TALome expansion occurred as a response to as yet unidentified *R* genes specific to *Xoc* (*Xo1* was not identified until 2015), or that TALome expansion in *Xoc* was associated with a lifestyle shift from a vascular to a parenchymal pathogen (i.e. instead of relying on SWEET genes, *Xoc* strains may need to simultaneously activate diverse sets of *S* genes to colonize the parenchyma). Additional factors probably played a role in determining TALome size in *Xoo* and *Xoc*, notably the genomic context of their TALEs differ in such a way that two different mechanisms have been proposed to play a role in TALE acquisition and mobility: a Tn*3*‐like transposition mechanism for *Xoo* and a integron‐like mechanism for *Xoc* (Erkes *et al*., 2017).

As the relationship between breeding and TALome size is not clear‐cut, breeding is probably not the only factor determining TALome size. Genome organization may play a crucial role because, of the groups that contain medium or large TALomes, with the exception of *Xcm*, all carry their TALEs exclusively in their chromosomes instead of plasmids. Genome organization is a factor that could indeed aid in the process of TALome expansion as genes are therefore less likely to be lost.

It is also possible that TALome expansion has occurred randomly in these groups, as a by‐product of genome duplications, and, so far, has not been selected against. However, this appears to be unlikely given that most TALEs in these groups have retained functional N‐ and C‐termini, and have not undergone extensive pseudogenization. Additional factors that may have contributed to TALome expansion, or a lack of it, include the presence/absence of other effectors or virulence mechanisms that might compensate for non‐virulent or avirulent TALEs, as well as the lifestyle of the bacteria: host range and tissue specificity. Rice, as a staple crop strongly affected by *Xanthomonas* species, has so far been at the centre of research into the biology of TALEs. Future genetic and genomic research in other host–pathogen systems will shed some light on how crop breeding feeds back on the evolution of a pathogen's attack arsenal.

This said, we believe that the diversity of strategies to counteract resistance against TALEs, found in *X. oryzae*, represents a cautionary tale as to what a large TALome can do against bred resistance. To overcome the highly adaptive potential of TALEs, future efforts in plant breeding programmes against *Xanthomonas* should focus on the use of a combination of strategies. With the advent of genome editing, synthetic promoters with target sequences for all CRDs in a given pathogen upstream of a single or multiple *R* genes could provide more durable resistance against a pathogen. Exploitation of resistance mechanisms that do not rely heavily on promoter activation, or loss of susceptibility through loss of TALE–promoter interactions, may provide an alternative approach. We postulate that pyramiding of a combination of resistance mechanisms [i.e. executor *R* gene promoter activation (*Xa7*, *Xa10*, *Xa23*, *Xa27*); loss of susceptibility (*xa13*, *xa25* and *xa41*) and traditional R protein recognition (*Xo1*/*Xa1*)], may provide a successful option for breeders to counteract pathogen evolution.
